# The annual recurrence of dengue in Bangladesh: A persistent threat

**DOI:** 10.1016/j.ijregi.2025.100835

**Published:** 2025-12-25

**Authors:** Tushar Ahmed Shishir, Nazifa Tabassum Tasnim, Akash Ahmed

**Affiliations:** Department of Mathematics and Natural Sciences, BRAC University, Dhaka, Bangladesh

**Keywords:** Dengue outbreak, Bangladesh, Dhaka, Vector-borne disease

## Abstract

This letter explores the annual recurrence of dengue infections in Bangladesh, a persistent threat to public health and well-being. We examine the cyclical nature of dengue infection across different regions, focusing on densely populated cities such as Dhaka and Chittagong. We also highlighted the challenges during the dengue epidemic by describing potential strategies for combating the spread, highlighting the severe health impacts on public health in high-prevalence areas such as Chittagong, Khulna, and Barisal. Recognizing dengue’s wide-reaching impact on health and daily life, we brainstorm the possible long and short-term solutions to prevent perpetuated future outbreaks.

## Highlights

• In 2023, dengue claimed 1705 lives and infected >321,000 individuals in Bangladesh.
• Young adults (aged 21-30 years) are most affected, with high fatality rates.
• Dhaka, Chattogram, Khulna, and Barishal reported the highest infection burden.
• Temporary vector control measure causes mosquito resistance.

## Graphical Abstract

**Figure:**
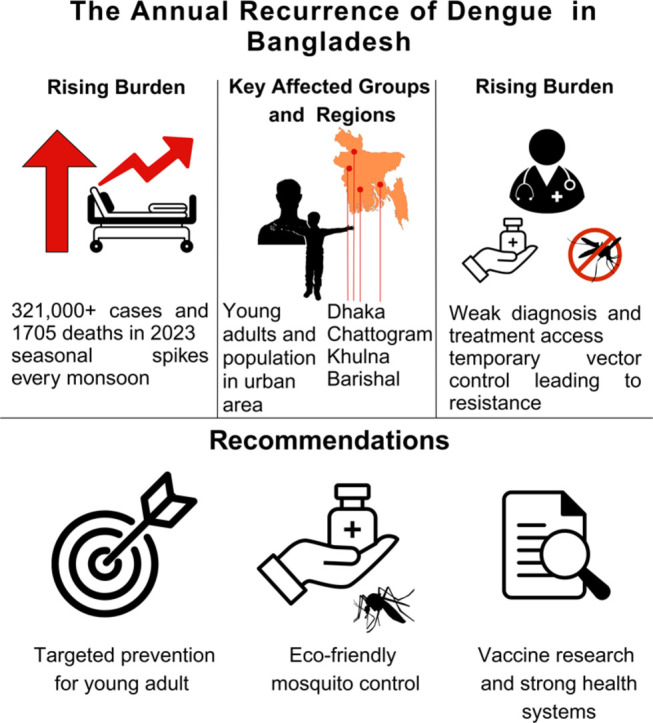

## Introduction

**Figure 1:**
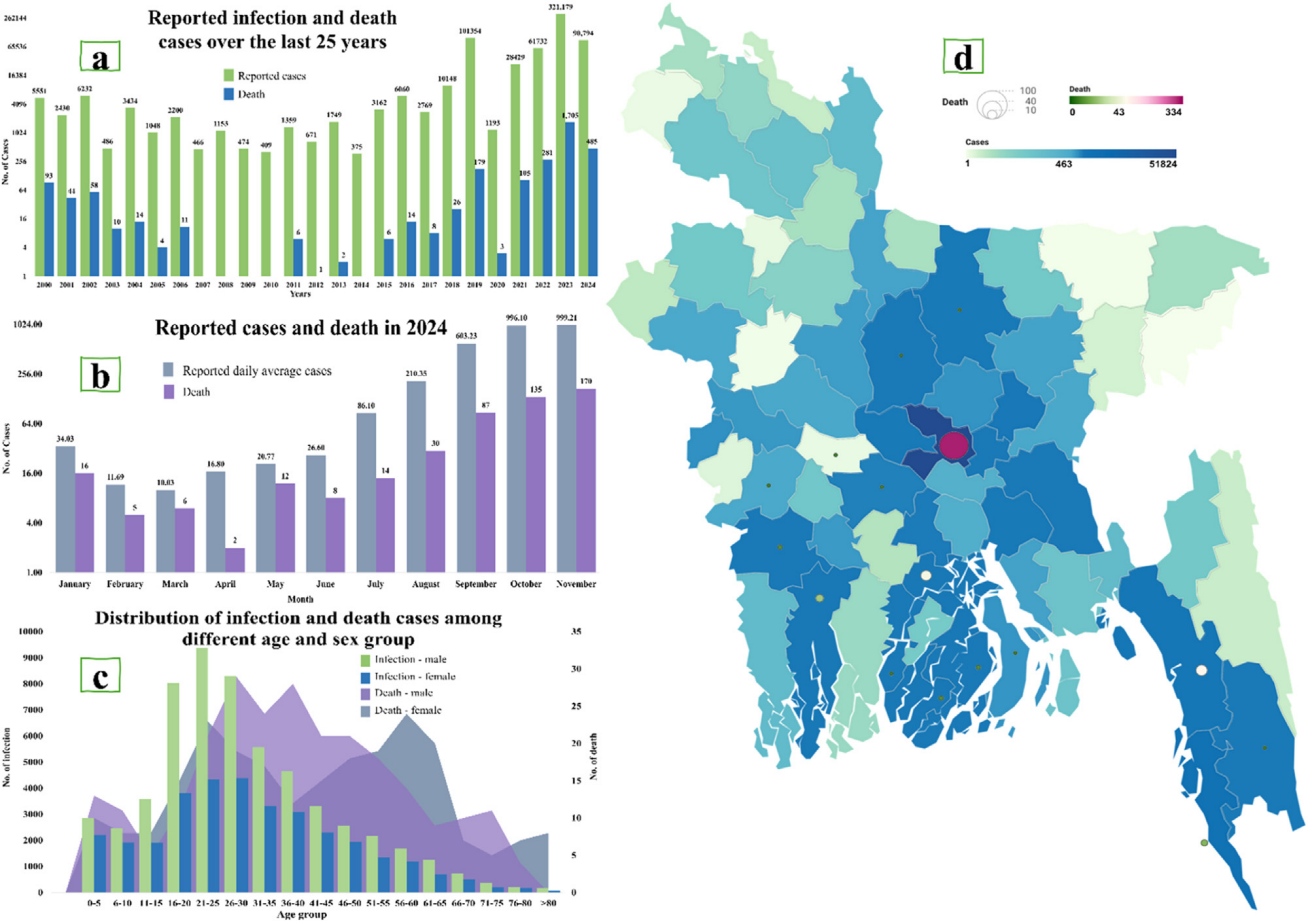
Geographical distribution and yearly trends of dengue in Bangladesh. (a) Reported infection and death cases over the last 25 years, (b) reported cases and deaths in 2024, (c) distributed infection and death cases among different age and sex groups, (d) geographical distribution of dengue cases in Bangladesh.

Every year, Bangladesh goes through the escalating battle against dengue fever, an illness transmitted by a vector *Aedes aegypti* and *Aedes albopictus* mosquito [1]. This viral infection has led to a public health emergency, causing thousands of cases annually, overwhelming the healthcare system in Bangladesh [2]. Bangladesh has experienced its deadliest dengue outbreak on record, with over 321,000 hospitalizations and 1705 deaths in 2023 alone. The crisis continues into 2024, with 61,817 cases, and 297 deaths already recorded by the end of October (Figure 1a). The severity of the situation calls for immediate action.

## The unforgiven cycle of dengue in Bangladesh

In Bangladesh, dengue is a recurring and yearly epidemic [3]. This year, the dengue cases in Bangladesh escalated sharply from mid-year, following the seasonal pattern observed in previous outbreaks, driven by the monsoon rains. Infections jumped from 26.60 cases in June to over 603.23 by September, with 87 deaths (Figure 1b). The outbreak even exaggerated in October, with 1024 cases and 128 deaths reported. Dhaka remains the hardest-hit area, with 51,824 cases and 334 deaths (Figure 1d). Other cities such as Chittagong, Khulna, and Barishal are also heavily affected because of their high population density and inadequate waste management, contributing to the rapid transmission of the virus.

Markedly, the country’s southern regions suffer more than the northern regions due to environmental differences [3]. Then again, the virus has hit men hardest, especially those aged 21-25 years, accounting for over 9384 cases, whereas young children, particularly, those aged 0-5 years, are also highly vulnerable, with 5052 reported cases. Deaths have been more common among young adults, with the highest fatality rates in the 21-25 and 26-30 years age groups. Women in the 26-30 years age group have been especially impacted, experiencing a higher number of deaths (Figure 1c).

## Challenges and recommendations in combating the dengue epidemic

Recent decades have seen a sharp rise in cases, with reported dengue infection, rising every monsoon season [4]. The current pattern is due to several factors, including Bangladesh’s warm and humid climate, increased rainfall, waste management, and the lack of mosquito control measures in populated air centers, especially in Dhaka and Chattogram [4]. To break the cycle of dengue, a mindful approach is needed, including integrating short- and long-term solutions. With the help of the government, local communities must prioritize mosquito control programs proactively by removing standing water, using insecticide-treated mosquito nets, and, most importantly, eco-friendly solutions of using larvivores fish in the harvesting water.

However, one of the main challenges while combating dengue is the lack of sustainable and effective mosquito control program. Although insecticide spray and fumigation are often used in affected areas, measures are temporary and can lead to mosquito resistance. In addition, limited diagnostic facilities, insufficient trained health care personnel, and poor accessibility to treatment centers future strain the overwhelmed health care system. Recent reports also indicated that young adults are distortional affected, requiring targeted prevention measure and awareness campaign for this age group [5].

## Dengue vaccine program and feasibility in Bangladesh

Several Asian countries, including Indonesia and Malaysia, have introduced dengue vaccination program using licensed vaccines such as Dengvaxia and Qdenga [6,7]. These programs aim to reduce severe dengue cases and hospitalization rates. However, in Bangladesh, the feasibility of implementing a nationwide dengue vaccination program remains challenging due to factors such as high vaccine cost, limited health care infrastructure, and the need for serotype-specific immunity assessment before vaccine [[8], [9], [10], [11]]. A potential vaccination strategy in Bangladesh would require targeted immunization of high-risk groups, such as young adults and residents of urban hotspots such as Dhaka and Chattogram, combined with strengthened vector control measures and public awareness campaigns to maximize effectiveness.

## Conclusion

The recent dengue outbreak in Bangladesh is a reminder of the pressing need for coordinated public health efforts, climate adaptation strategies, and international support. If not handled properly, the virus will continue to intensify and escape the system. Although the World Health Organization and other organizations provide valuable assistance, more work is required, including thorough research and the acquisition of vaccines, to achieve a long-term solution. In addition, improving vector control and fortifying public health infrastructure are essential for providing immediate relief.

## Declaration of competing interest

The authors have no competing interests to declare.
